# Gitelman syndrome with Graves’ disease leading to rhabdomyolysis: a case report and literature review

**DOI:** 10.1186/s12882-023-03180-8

**Published:** 2023-05-02

**Authors:** Jing Xu, Juan He, Shujing Xu, Rui Wang, Nianchun Peng, Miao Zhang

**Affiliations:** Department of Endocrinology and Metabolism, the Affiliated Hospital of Guizhou Medical University, Guiyang, 550004 China

**Keywords:** Gitelman syndrome, Graves’ disease, Rhabdomyolysis, The *SLC12A3* gene, Case report

## Abstract

A 14-year-old male patient who suffered from limb numbness, fatigue, and hypokalemia was considered Graves’ disease (GD) complicated with thyrotoxic periodic paralysis (TPP) at the first diagnosis. Although with the treatment of antithyroid drugs, he developed severe hypokalemia and rhabdomyolysis (RM). Further laboratory tests revealed hypomagnesemia, hypocalciuria, metabolic alkalosis, hyperrenin, and hyperaldosteronemia. Genetic testing revealed compound heterozygous mutations in the *SLC12A3* gene (c.506-1G > A, c.1456G > A) encoding the thiazide-sensitive sodium-chloride cotransporter, which presented a definitive diagnosis of Gitelman syndrome (GS). Moreover, gene analysis revealed his mother diagnosed with subclinical hypothyroidism due to Hashimoto’s thyroiditis carried the c.506-1G > A heterozygous mutation in the *SLC12A3* gene and his father carried the c.1456G > A heterozygous mutation in the *SLC12A3* gene. His younger sister who had hypokalemia and hypomagnesemia carried the same compound heterozygous mutations as the proband and was diagnosed with GS as well, but with a much milder clinical presentation and better treatment outcome. This case suggested the potential relationship between GS and GD, clinicians should strengthen the differential diagnosis to avoid missed diagnosis.

**Supplementary Information:**

The online version contains supplementary material available at 10.1186/s12882-023-03180-8.

## Introduction

Gitelman syndrome (GS) is a rare autosomal recessive salt-losing tubulopathy mainly characterized by hypokalemia, hypomagnesemia, hypocalciuria, and metabolic alkalosis [1]. The prevalence of GS is about (1–10)/40,000, and may be higher in Asian populations. Homozygous or compound heterozygous mutations in the *SLC12A3* gene encoding the thiazide-sensitive sodium-chloride cotransporter located in the distal convoluted tubule of the kidney have been identified in the pathogenesis of GS [2]. In addition to GS, the causes of hypokalemia also include a variety of endocrine diseases, such as hyperthyroidism, Cushing syndrome, primary aldosteronism, etc. Thyrotoxic periodic paralysis (TPP) is easily confused with GS, leading to missed diagnosis, misdiagnosis, and delayed treatment. Here we reported a patient who was initially diagnosed with TPP, but recurrently presented hypokalemia even induced severe rhabdomyolysis (RM) after the control of hyperthyroidism. After some relevant laboratory tests and genetic testing, the final diagnosis was Graves’ disease (GD) combined with GS.

## Case presentation

**Fig. 1:**
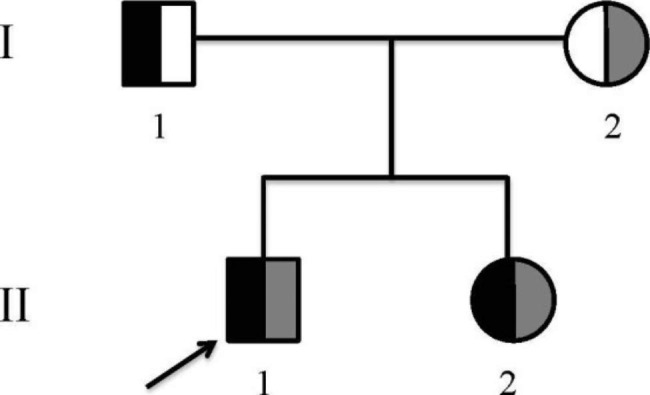
The Gitelman syndrome family (I_1_, the proband’s father; I_2_, the proband’s mother; II_1_, the proband; II_2_, the proband’s sister; the black legend, c.1456G > A; the grey legend, c.506-1G > A).

The patient was a 14-year-old male (the proband, II1) with a 5-month history of recurrent limb numbness and muscle weakness. 5 months ago, the patient was diagnosed with hypokalemia due to weakness of limbs in the local hospital and was referred to the emergency department of our hospital. After the relevant examination (Table 1), he was diagnosed with “GD with TPP”, and was treated with potassium supplementation, methimazole, and propranolol. On the admission to our department, he had been suffering from severe limb numbness, fatigue, paroxysmal tetany, abdominal pain, and vomiting for a day. Physical examination showed the blood pressure was 123/59 mmHg, pulse was 94 beats/minute, height was 154.5 cm (normal), weight was 58 kg (+ 2 SDs), and body mass index (BMI) was 24.3 kg/m^2^. There was no growth retardation, no prominent bulging of the eyes, and 2 degrees of diffuse thyroid enlargement existed without nodules. The patient denied a history of hypertension and the use of any potassium-lowering drugs. His parents were not in consanguineous marriage. His mother had a history of hypothyroidism, and his 9-year-old sister had a history of hypokalemia after an upper respiratory tract infection (Fig. 1).

**Table 1:** Laboratory parameters of the proband

	First visit	This visit Day 1	This visit Day 3	This visit Day 7	This visit Day 14	This visit 1month	This visit 3 month	Reference
medication	-	methimazole, propranolol	methimazole, potassium	methimazole, potassium, magnesium sulfate, calcium gluconate	methimazole, potassium magnesium aspartate, potassium chloride	methimazole, potassium magnesium aspartate, potassium chloride	methimazole, potassium magnesium aspartate, potassium chloride (refuse to add spironolactone)	-
K (mmol/L)	2.408	2.297	3.470	4.370	3.600	3.000	2.940	3.500–5.300
Na (mmol/L)	142.39	137.63	140.29	142.89	142.59	141.46	141.28	137.00-147.00
Cl (mmol/L)	98.83	96.24	99.33	96.14	96.08	96.39	98.63	96.00-108.00
Ca (mmol/L)	1.993	2.016	1.950	2.430	2.540	2.300	2.230	2.250–2.670
Mg (mmol/L)	0.568	1.101	0.670	0.610	0.540	0.590	0.620	0.750–1.020
P (mmol/L)	1.240	0.850	1.110	1.640	0.720	1.210	1.290	0.95–1.65
HCO3^−^ (mmol/L)	23.80	22.70	24.70	30.20	32.80	31.90	28.30	23.00–30.00
FT3 (pmol/L)	8.47	5.10	-	-	-	7.20	7.99	3.93–7.70
FT4 (pmol/L)	38.45	18.65	-	-	-	17.77	15.12	12.60–21.00
TSH (IU/L)	0.006	0.011	-	-	-	0.864	2.130	0.510–4.300
TRAb (U/ml)	2.93	1.03	-	-	-	1.32	2.36	0-1.75
TPOAb (U/ml)	118.00	60.34	-	-	-	-	-	0–34.00
TGAb (U/ml)	21.56	12.91	-	-	-	-	-	0-115.00

Partial results of laboratory tests are detailed in Table 1. Other initial laboratory analysis showed the following: 24-hour urinary potassium, 158.33 mmol (normal range 25.00-100.00); 24-hour urinary calcium, 0.288 mmol (normal range 2.5–7.5); 24-hour urinary magnesium, 6.80 mmol (normal range 3.00–5.00); 24-hour urinary chlorine, 245.59 mmol (normal range 170.00-250.00); Serum creatinine, 105.79 umol/L (normal range 57.00–97.00); Uric acid, 1435.30 umol/L (normal range 208.00-428.00); Creatine kinase (CK), 31,567 U/L (normal range 50–310); CK Isozyme, 564 U/L (normal range 0–25); troponin T, 0.073 ng/ml (normal range 0-0.014); myoglobin, 1416 ng/ml (normal range 28–72). Blood gas analysis revealed pH, 7.453 (normal range 7.350–7.450) and bicarbonate, 37.60 mmol/L (normal range 21.40–27.30). Renin activity, 13.98 ng/ml/hr (normal range 1.31–3.95), aldosterone was 449.76 pg/ml (normal range 40.00-310.00), ARR was 3.22. His liver function, gonadal steroid hormones, and the circadian rhythm of plasma cortisol were normal. 1 mg of dexamethasone suppression test was negative. Electrocardiogram showed sinus rhythm, heart rate 91 bpm, and high voltage of the left ventricular (Fig. S1). Abdominal computed tomography (CT) showed intestinal stasis, no abnormalities in the adrenal glands or kidney (Fig. S2).

**Fig. 2:**
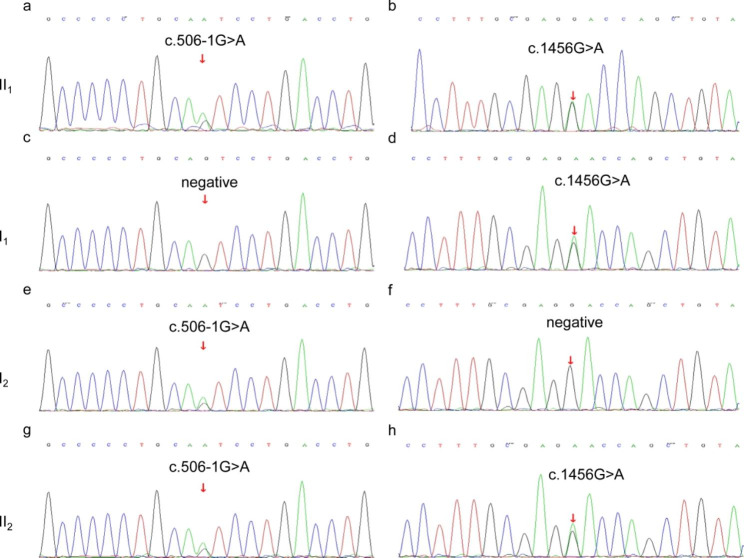
Sanger sequencing verification of the *SLC12A3* gene in this family (a. c.506-1G > A, heterozygous mutation in intron 3 of proband II_1_; b. c.1456G > A, heterozygous mutation in exon 12 of proband II_1_; c. no mutation in intron 3 of I_1_; d. c.1456G > A, heterozygous mutation in exon 12 of I_1_; e. c.506-1G > A, heterozygous mutation in intron 3 of I_2_; f. no mutation in exon 12 of I_2_; g. 506-1G > A, heterozygous mutation in intron 3 of II_2_; h. c.1456G > A, heterozygous mutation in exon 12 of II_2_; II_1_, the proband; I_1_, the proband’s father; I_2_, the proband’s mother; II_2_, the proband’s sister)

After obtaining the patient’s, his parents’ and his younger sister’s consent, peripheral blood samples were collected and sent to the Maikino Medical Laboratory (Beijing, China). The *SLC12A3* gene was detected by the whole exome sequencing and verified by the Sanger sequencing. The proband (II_1_) carried compound heterozygous mutations in the *SLC12A3* gene (Fig. 2a, b). One originating from his father is a known heterozygous missense mutation (c.1456G > A) located in exon 12, resulting in the change of the amino acid from aspartic acid to asparagine (p.D486N) (Fig. 2c, d) [3]. The other originating from his mother is also a known heterozygous mutation (c.506-1G > A) located in intron 3, resulting in a splicing mutation of the amino acid (Fig. 2e, f) [4]. c.1456G > A was predicted to be harmful by PolyPhen-2, Mutation Taster, and SIFT. Moreover, both mutations are classified as pathogenic according to American College of Medical Genetics (ACMG) criteria. His younger sister (II2) carried the same compound heterozygous mutations (Fig. 2g, h), who was diagnosed with GS as well. The laboratory parameters of other family members were shown in Table 2. I_1_ was diagnosed with subclinical hypothyroidism, and I_2_ was diagnosed with primary hypothyroidism due to Hashimoto’s thyroiditis.

**Table 2:** Laboratory parameters of other family members

	I_1_	I_2_	II_2_ (before treatment)	II_2_ (1 week)	II_2_ (1 month)	Reference
medication	-	-	-	potassium magnesium aspartate	potassium magnesium aspartate	-
Age (year)	39	36	9	9	9	-
K (mmol/L)	4.010	4.690	3.470	3.54	3.720	3.500–5.300
Na (mmol/L)	140.55	139.30	142.32	142.99	140.42	137.00-147.00
Ca (mmol/L)	2.260	2.270	2.260	2.360	2.420	2.250–2.670
Mg (mmol/L)	0.840	0.880	0.520	0.539	0.610	0.750–1.020
P (mmol/L)	1.080	1.060	1.260	1.550	1.560	0.95–1.65
HCO3^−^ (mmol/L)	22.70	22.90	27.70	25.70	23.60	23.00–30.00
FT3 (pmol/L)	5.82	4.59	7.70	-	-	3.93–7.70
FT4 (pmol/L)	15.79	10.78	19.76	-	-	12.60–21.00
TSH (IU/L)	4.80	10.303	3.900	-	-	0.510–4.300
TRAb (U/ml)	0.81	1.48	1.49	-	-	0-1.75
TPOAb (U/ml)	6.94	208.70	6.95	-	-	0–34.00
TGAb (U/ml)	13.01	109.00	12.25	-	-	0-115.00

I_1_, the proband’s father; I_2_, the proband’s mother; II_2_, the proband’s sister

The proband was given potassium supplementation (potassium chloride, at least 15 g/d), adequate hydration, alkalization of urine, and diuresis. The CK, CK isoenzymes, troponin T, myoglobin and creatinine gradually returned to normal. The follow-up therapies included potassium chloride 3 g/d, and potassium and magnesium aspartate 9 tablets/d. After 3 months, hypokalemia and hypomagnesemia of the proband were still difficult to be corrected. The addition of spironolactone was recommended, but he refused (Table 1). For the treatment of GD, methimazole 2.5 mg/d orally was given (Table 1). His sister was also given potassium and magnesium aspartate 9 tablets/d. After 1 month, she showed normal potassium and low magnesium levels (Table 2). These two patients are still under follow-up treatment.

## Discussion

Common clinical causes of hypokalemia include insufficient intake, increased excretion, and intracellular transfer of potassium. As the proband had no symptoms including frequent vomiting, long-term diarrhea, and profuse sweating, hypokalemia caused by reduced intake and increased excretion of potassium from the digestive tract and skin was not considered. Due to abnormal thyroid function and hypokalemia, the proband was considered GD with TPP at the initial diagnosis. Hypokalemia caused by GD was due to the intracellular transfer of potassium via increased activity of Na+-K+-ATPase in the cell membrane [5]. The proband had a well-controlled thyroid function under the treatment of methimazole, but still presented persistent hypokalemia which could not be explained by TPP alone. The patient had normal blood pressure and no history of potassium-affecting drugs such as licorice or diuretics. In combination with his history and laboratory findings, GS or Bartter syndrome (BS) was highly suspected diagnostically. BS is a group of autosomal recessive renal tubular diseases manifested by hypokalemia, renal salt loss, metabolic alkalosis, elevated renin and aldosterone levels, and normal blood pressure. However, BS usually has an onset in early childhood with growth retardation [6]. The laboratory tests usually present normal serum magnesium levels and mild hypercalciuria, which were inconsistent with our patient. Besides, previous study reported that patients with HNF1B-related nephropathy can have hypokalemia and hypomagnesemia. However, patients may present renal cysts and diabetes [7], which were inconsistent with our patient as well. Moreover, further genetic testing of hypokalemia-related genes revealed reported pathogenic compound mutations in the *SLC12A3* gene, so the clinical diagnosis of GS can be identified.

So far, more than 500 *SLC12A3* gene mutations have been reported. Over 70% of GS patients were compound heterozygous mutations. Four missense mutations including Thr60Met, Asp486Asn, Arg913Gln, and Arg928Cys, had a high-frequency allele frequency of > 3% in Chinese patients [8]. The proband and his younger sister in this case carried Asp486Asn mutation (p.D486N). To date, studies have reported some correlation between clinical phenotype and genotype of GS [9], for example, Thr60Met may be associated with earlier onset and lower urinary calcium excretion in Chinese pediatric GS [10]. Some intronic mutations such as c.506-1G > A, were associated with more severe hypokalemia [11], consistent with our proband who carrying the same intronic mutation. Moreover, the severity of GS is closely related to gender differences. Previous studies indicated that female GS patients had milder clinical symptoms than male GS patients even if the mutations were identical [12, 13]. This feature was also observed in our case. The potential mechanism is still unclear but may be related to estrogen levels. As estrogen can enhance the density of thiazide-sensitive sodium chloride cotransporter in rat distal convoluted tubule [14], which may partly maintain electrolyte balance. However, cases were limited to draw firm conclusions.

GS can be combined with thyroid disease. A Japanese study showed that approximately 4.3% of GS patients had a combination of abnormal thyroid function [15]. Table 3 summarized the reported cases of GS diagnosed by genetic testing with co-morbid thyroid diseases. Almost all such patients were from East Asian populations, presumably associated with the high incidence of GS and autoimmune thyroid disease per se in this area. At present, there is no definite evidence of an association between the *SLC12A3* gene and the pathogenesis of thyroid disease. In a previous study of a family with GS complicated with thyroid disease, it was found that the thyroid function was abnormal in members carrying the c.1456 G > A heterozygous mutation (p.D486N) in exon 12 of the *SLC12A3* gene, regardless of whether or not they had a diagnosis of GS, leading to the hypothesis that the mutation at this locus may affect thyroid function [3]. However, it should be noted that this mutation site itself is a high-frequency mutation site in the *SLC12A3* gene in the Chinese population, and the current sample size is small. Whether this mutation site is related to thyroid dysfunction in GS patients still needs to be confirmed by further studies. A previous study have confirmed that iodine metabolism is closely related to magnesium metabolism [16]. Other studies also demonstrated that low magnesium may cause the recurrence of hyperthyroidism [17], while magnesium supplementation can promote the normalization of thyroid morphology and function [18]. These evidences partly explain the relationship between GD and GS.

**Table 3:** Cases based on genetic diagnosis of GS with thyroid disease

Age (y)	Gender	Nationality	Complication	Mutation type	Mutation site	Mutation of amino acid	Reference
18	female	Japan	Graves’ disease	compound heterozygous	-	Thr339Pro/Leu858His	[26]
50	female	Japan	Graves’ disease	compound heterozygous	-	Thr180Lys/349Ser	[26]
56	female	Japan	Graves’ disease	homozygous	-	Ala569Val	[26]
16	male	China	Graves’ disease	compound heterozygous	1456G > A/2102_2107 delACAAGA	Asp486Asn/701_702delAsnLys	[3]
10	female	China	elevated FT3, TSH	compound heterozygous	1456G > A/2102_2107 delACAAGA	Asp486Asn/701_702delAsnLys	[3]
45	male	China	Graves’ disease	heterozygous	1562_1564delTCA	522delIle	[27]
21	male	Japan	Graves’ disease	compound heterozygous	539 C > A/2537T > A	Thr180Lys/Leu858His	[28]
2	male	China	Graves’ disease	compound heterozygous	1077 C > G/1567G > A	Asn359Lys/Ala523?	[29]
42	female	China	Hashimoto’s thyroiditis, subclinical hypothyroidism	heterozygous	248G > A	Arg83Gln	[30]
18	female	USA	Pregnancy-related thyrotoxicosis	homozygous	2581 C > T	Arg861Cys	[31]
40	female	Japan	Hashimoto’s thyroiditis	compound heterozygous	2552T > A/2561G > A	Leu849His/Arg852His	[25]
28	female	Japan	Graves’ disease	homozygous	2552T > A	Leu849His	[25]
14	female	China	Graves’ disease	homozygous	791G > C	Gly264Ala	[32]
46	female	China	Graves’ disease	heterozygous	185 C > T	Thr60Met	[33]
21	male	China	Graves’ disease	homozygous	2744G > A	Arg913Gln	[33]
39	male	China	Graves’ disease	compound heterozygous	1841 C > T/2968G > A	Ser614Phe/Arg990Lys	[34]
41	female	China	Hashimoto’s thyroiditis	compound heterozygous	964 + 2T > C/179 C > T	Ala285ArgfsX48/Thr60Met	[34]

Rhabdomyolysis (RM) is a syndrome caused by the breakdown of skeletal muscle fibers, resulting in the release of intracellular substances into the systemic circulation. Typical laboratory tests of RM show significantly elevated CK more than 4 times the upper limit of normal and abnormal renal function. Muscle trauma is the most common cause of RM, other causes include excessive physical exertion, inherited metabolic diseases, infectious diseases, drugs (e.g. licorice), alcohol, and electrolyte abnormalities (especially hypokalemia) [19]. Potassium is crucial for vasodilation and muscle contraction. Hypokalemia-induced muscular ischemia may increase the permeability of cell membrane, raising the intracellular calcium concentration to destroy myofibrillar, cytoskeletal and membrane proteins, thus leading to muscle necrosis and intracellular CK and myoglobin released into the blood circulation [20]. In this case, the patient was admitted with severe hypokalaemia and tetany which can lead to rhabdomyolysis. Also, some patients with hyperthyroidism can develop RM, which may be related to the increased consumption of energy and substrate stores in muscles [21]. However, our proband developed RM under the stable control of thyroid function. Given the clear diagnosis of GS, we speculated RM was due to persistent hypokalaemia associated with GS but not GD. Although hypokalemia is a common clinical manifestation of GS, GS complicated with RM is relatively rare [22, 23]. Compared to the patients of GS alone, the mutation type and site of the *SLC12A3* gene in GS with RM patients are not specific.

The treatment of GS should be individualized. Lifelong potassium supplementation, usually potassium chloride, is necessary. In the setting of concomitant hypomagnesemia, magnesium supplementation should be considered first because it reduces urinary potassium excretion and reduces the risk of tetany and other complications. The ideal target is 3.0 mmol/L for serum potassium and 0.6 mmol/L for serum magnesium. During treatment, it should be noted that high-dose supplementation may cause side effects such as gastric ulcer, vomiting or diarrhea, and electrolyte imbalance [1]. If hypokalemia and low magnesium are persistently difficult to correct, combined medication should be considered, including spironolactone and angiotensin-converting enzyme inhibitors (ACEIs) or angiotensin receptor blockers (ARBs) [1]. In addition, studies have demonstrated that the cyclooxygenase-2 (COX-2) inhibitor rofecoxib can rapidly increase serum potassium concentration, inhibit high renin and aldosterone activity, and improve RM [24]. In this study, the treatment for the proband was mainly divided into three parts: (1) the treatment for RM and acute kidney injury. GS induced hypokalemia was considered as the cause of severe RM, so the supplementation of potassium chloride and magnesium was prioritized. In addition, active rehydration and urine alkalinization were crucial for the correction of abnormally elevated creatinine, CK and myoglobin. (2) Long-term therapy of low potassium and low magnesium for GS. Intravenous and oral supplementation of potassium and magnesium were used, and the dose of medication was adjusted according to the electrolyte results. At the 3-month follow-up, the serum magnesium of the proband could be maintained at 0.6 mmol/L, but the serum potassium was still lower than 3.0 mmol/L. The patient was advised to add spironolactone, but he refused (Table 1). (3) Antithyroid therapy for Graves’ disease. The previous study showed that excessive thyroid hormone may lead to increased renal excretion of electrolytes, thereby aggravating the clinical symptoms of GS patients [25], so maintaining stable thyroid function was critical. The proband was regularly treated with methimazole after admission and was well controlled during the 3-month follow-up (Table 1). Clinical symptoms of GS patients are highly heterogeneous. As in this case, even though the two patients had the same gene mutations, the clinical features and treatment outcomes were varied. Clearly, the potassium and magnesium supplementation of his sister was more effective (Table 3).

## Conclusions

There are multiple causes of hypokalemia. With the popularity of genetic screening technology, more and more cases of GD combined with GS have been identified in recent years. However, the symptoms and signs caused by GS are easily masked by TPP, making it easy to miss the diagnosis clinically. Therefore, when patients showed refractory hypokalemia, in addition to family history, blood and urine electrolytes, renin-angiotensin system, and other evaluations, genetic testing is necessary for the diagnosis to avoid missed diagnosis and misdiagnosis. The specific relationship between GS and GD is still unclear. Some special GS gene mutation sites may be related to the pathogenesis of thyroid diseases, and hypomagnesemia caused by GS may be closely related to the recurrence of GD.

## Electronic supplementary material

Below is the link to the electronic supplementary material.

Supplementary Material 1

## Data Availability

The raw data supporting the conclusions of this article will be made available by the corresponding author without undue reservation.

## Abbreviations

GS: Gitelman syndrome
GD: Graves’ disease
TPP: thyrotoxic periodic paralysis
RM: rhabdomyolysis
CK: Creatine kinase
ARR: aldosterone/renin ratio
BS: Bartter syndrome
ACEIs: angiotensin-converting enzyme inhibitors
ARBs: angiotensin receptor blockers
COX-2: cyclooxygenase-2
