# Supplementary material for: Gitelman syndrome with Graves’ disease leading to rhabdomyolysis: a case report and literature review
Source: BMC Nephrol. 2023 May 2;24:123. doi: 10.1186/s12882-023-03180-8 (PMC10152583; doi:10.1186/s12882-023-03180-8)
Supplement: Supplementary file 1 — Supplementary Material 1 [file 12882_2023_3180_MOESM1_ESM.docx]

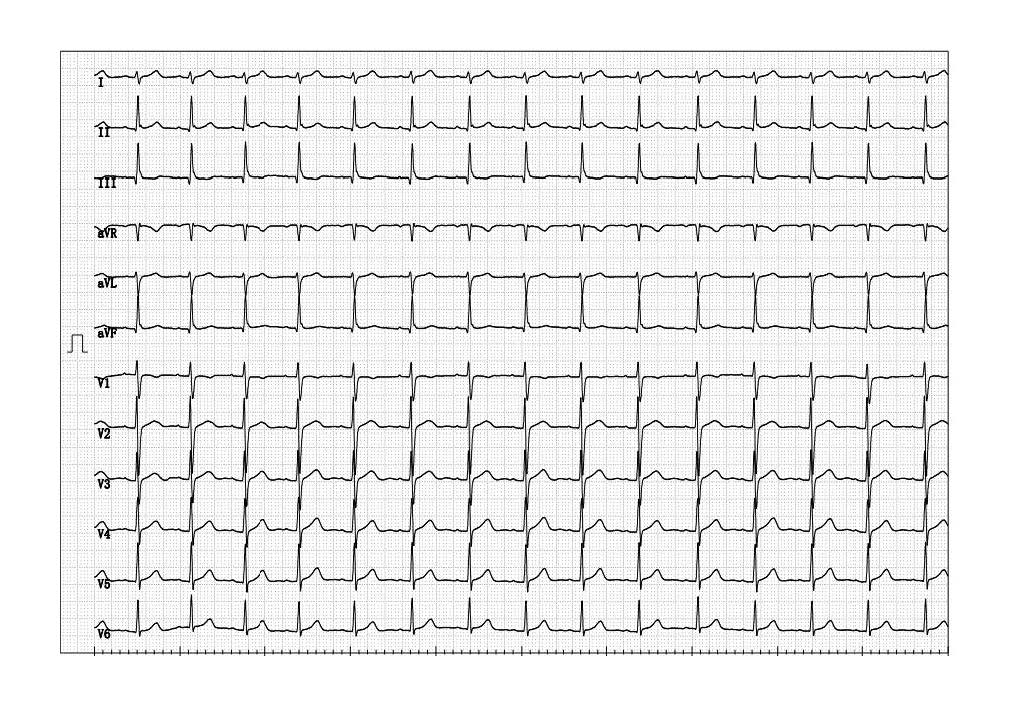


**Fig. S1** Electrocardiogram of the proband showed sinus rhythm, heart rate 91 bpm, and high voltage of the left ventricular.


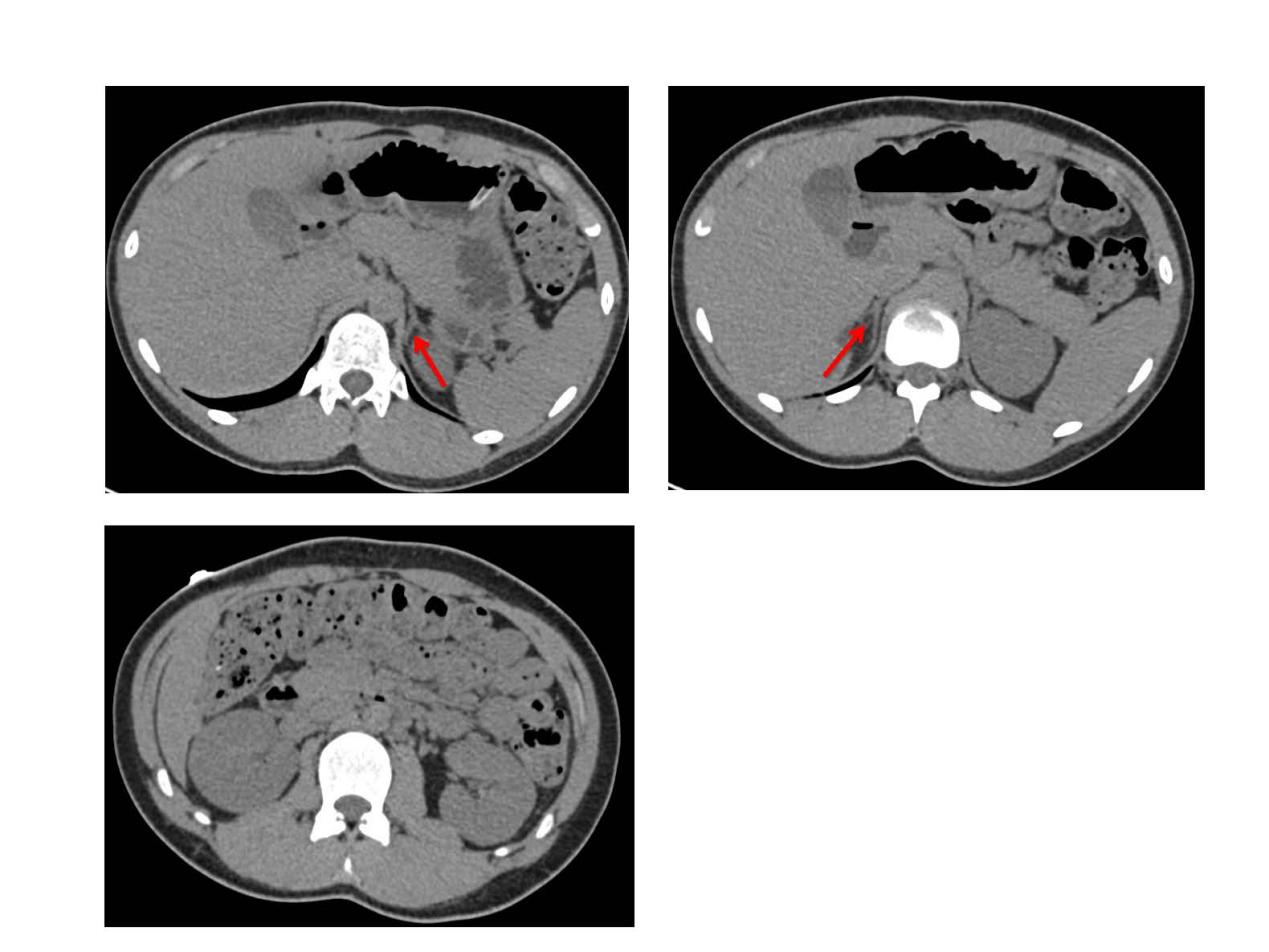


**Fig. S2** Abdominal CT of the proband showed intestinal stasis, no abnormalities in the adrenal glands (the red arrow) or kidney.
